# Global school-based student health survey: country profiles and survey results in the eastern Mediterranean region countries

**DOI:** 10.1186/s12889-022-12502-8

**Published:** 2022-01-19

**Authors:** Elham Abdalmaleki, Zhaleh Abdi, Sahand Riazi Isfahani, Sara Safarpoor, Bahar Haghdoost, Saharnaz Sazgarnejad, Elham Ahmadnezhad

**Affiliations:** 1 National Institute of Health Research (NIHR), Tehran University of Medical Sciences, Tehran, Iran; 2 School of Medicine, Tehran University of Medical Sciences, Tehran, Iran

**Keywords:** Global school-based student health survey, Adolescence, Eastern Mediterranean region, Sustainable development goals, Universal health coverage, Non-communicable diseases

## Abstract

**Background:**

The increasing prevalence of non-communicable diseases (NCDs) has some major implications on many countries to achieve universal health coverage. This study aimed to investigate the implementation of Global School-based Student Health Survey (GSHS), which is used to assess the risk factors of NCDs among children and adolescents in the eastern Mediterranean region (EMR).

**Methods:**

This study was a meta-analysis and systematic literature review of 2001–2018 published studies, which were found by searching PubMed, Google Scholar, WHO, and CDC databases. In this study, the target group was students aged between 13 and 17 years old. GSHS implementation as well as risk factors of NCDs were compared across different countries. The random-effect model for meta-analysis was considered at 95% confidence interval.

**Result:**

In the EMR, 19 countries have implemented GSHS at least once following the survey manual (37 surveys). Overall, 201,795 students were included in our analysis. The overall estimation prevalence rateof the overweight was 24.5% (20.6–28.8), obesity was 7.3% (5.4–9.5), insufficient physical activity was 82.4% (80.7–84.1), tobacco usage was 14.3% (10.53–18.67), and smoking was 9.6% (8.1–11.3), respectively. Among those aged 13 to 17 years old, these rates were estimated as 19.8 (13.2–27.3), 9.7 (6.2–14.0), 86.1 (84.1–87.9), 17.8 (11.8–24.7), and 11.5 (9.4–13.8), respectively.

**Conclusion:**

GSHS has been widely implemented across EMR countries. Using nationally representative data, the results show that more efforts are needed to target the NCDs risk factors among adolescents in the region.

**Supplementary Information:**

The online version contains supplementary material available at 10.1186/s12889-022-12502-8.

## Background

Nearly 35% of the global burden of diseases originates in adolescence, and more than 3000 adolescents die every day, mostly due to Non-communicable diseases (NCDs), injuries, and other preventable causes [1]. The Eastern Mediterranean Region (EMR) suffers from high prevalence rates of NCD-related risk factors [2]. The World Health Organization (WHO) in collaboration with other UN agencies and technical assistance of the Centers for Disease Control and Prevention (CDC) introduced Global School-based Student Health Survey (GSHS) in 2001, which has been started around the world since 2003. The purpose of GSHS is providing accurate data on health behaviors and protective factors among students, in order to help countries develop more priorities; establish appropriate programs; advocate for resources for school health programs and policies; and allow international agencies and countries to make comparisons across countries [3]. The GSHS is a rapid, affordable, useful tool and a suitable alternative for surveillance systems [4, 5]. Of note, the GSHS is a part of the WHO STEPwise approach to Surveillance [4]. Correspondingly, this approach has three steps for data collection, including performing the questionnaire-based assessment, biochemical measurements, and laboratory measurements [6]. In this regard, it is recommended to conduct GSHS every 3 to 5 years [7]. This survey uses a standardized scientific sample selection process, the common school-based methodology, core questionnaire modules, and three questionnaires (including co core questionnaire modules, core-expanded questions, and country-specific questions, which are combined to form a country-specific self-administered questionnaire) [8]. Accordingly, the 10 core questionnaire modules address the leading causes of morbidity and mortality, including alcohol abuse, dietary behaviors, drug abuse, hygiene, mental health status, physical activity, protective factors, sexual behaviors, tobacco usage, violence, and unintentional injury, among children and adults worldwide. Countries must select at least six of the 10 core modules in their country-specific questionnaire [3, 9]. Thereafter, country-specific questions can be added to the GSHS core questionnaire [10]. About, 99 countries have implemented this survey [11]. The GSHS results could help in guiding policy makers to plan, administer, and implement some interventions targeting the NCD risk factors in adolescents [12]. Following the introduction of the Sustainable Development Goals (SDG) in 2015, monitoring the interventions to reduce NCDs and the related risk factors globally received increasing attention [13, 14]. Therefore, the appropriate implementation of such surveys is of great importance [15]. This study aimed at examining the implementation status of GSHS in the EMR, comparing prevalence rates of NCD risk factors, including alcohol and tobacco abuse, obesity and overweight (inappropriate dietary behaviors), and physical activity among EMR countries as well as estimating an overall value for each one of these indicators in adolescents aged between 13 and 15 and between 13 and 17 years old, using the meta-analysis method.

## Methods

This study was a systematic review and meta-analysis conducted in two consecutive phases. The first phase was the comparison of the surveys’ designs, including questionnaires, modules, tools, and the implementation period. In addition, the second phase was the comparison of the results of these surveys, including the indicators for tobacco usage (smoking and consumption of any type of tobacco), alcohol use, obesity and overweight (inappropriate nutritional behaviors), and physical activity in terms of gender in 22 EMR countries. Afterward, the results obtained for each indicator were combined to estimate an overall value for all member states using a meta-analysis design. Prior to 2012, GSHS was only performed among students aged between 13 and 15 years old, but since 2013, the age of the target group has changed to people aged between 13 and 17 years old. Accordingly, these two age groups were compared among EMR countries. For the countries that implemented GSHS once before and once after 2013, the results were presented for both age groups of 13–15 and 13–17 years old. However, the ages of the participants investigated in the studies performed in Iran and Saudi-Arabia were different from those of other countries in other studies. Accordingly, to preserve the consistency, their indicators were considered in the range of 13 to 17 years old.

### Phase 1- comparison of surveys conducted across the EMR countries

**Fig. 1:**
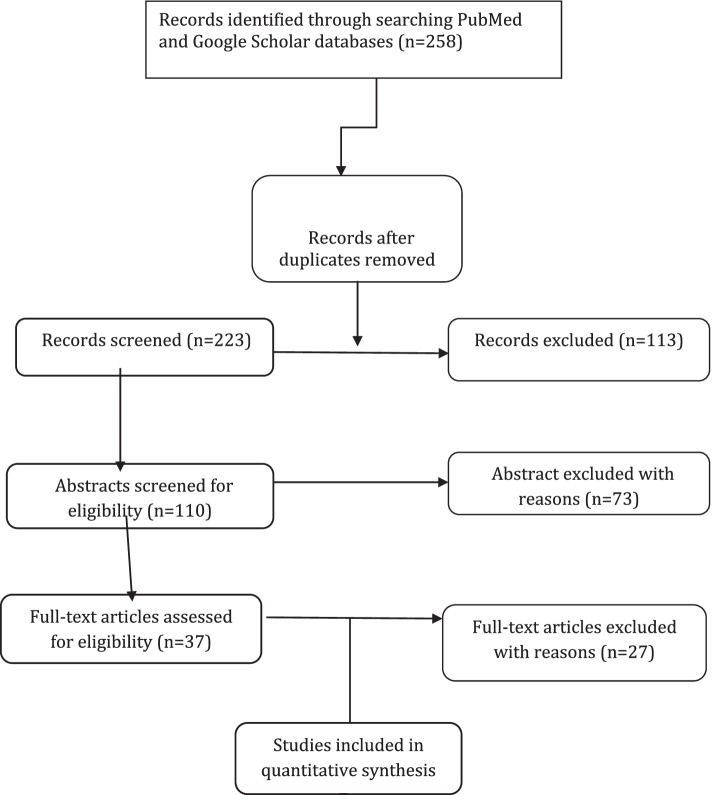
PRISMA Flowchart. The PRISMA flow diagram for the review detailing the database searches, the number of abstracts screened, and the full texts retrieved

The WHO and CDC databases were the main information sources for this study. In cases where there was no data from member states published on these databases, other databases such as PubMed and Google Scholar, were searched. Keywords used for this search included: “Global School Health Survey (GSHS)”, “Non-communicable disease Prevention”, “school-based survey”, “Childhood and Adolescence Surveillance”, “Protocol”, and “methodology” along with the name of the countries with different combinations of search terms in the subjects and abstracts. Figure 1 shows PRISMA flow diagram, summarizing the review process [1]. Of note, the published surveys reports were not included in the process.

The surveys were compared in terms of the modules, their sample sizes, and response rate using published country reports and factsheets between 2001 and 2018 by the WHO [11]. Information of Iran and Saudi-Arabia surveys were extracted from the published articles. Moreover, the questionnaires and modules were compared across the EMR countries. Finally, core modules and their frequencies of execution were compared across all these included surveys.

### Phase 2-comparison of the surveys’ results

The WHO dataset was used as the principal data source to examine the survey results for each country. The indicators of the Saudi-Arabia and Iran studies were extracted from both articles and national reports. The survey’s results were combined using a random-effect model of meta-analysis. Thereafter, the statistical heterogeneity was examined using I^2^ statistic and the statistical significance level was considered as *P*-value < 0.05. Due to the high heterogeneity among the studies, the random-effect model was used to combine the results at 95% confidence interval. For performing statistical analyses, the variance of each study was initially calculated using binomial distribution variance, by considering the binomial distribution of the prevalence rate. The weight given to each study was proportional to the inverse-variance. The included studies were combined in terms of the number of samples and variance. All these analyses were performed using metaprop command in STATA16 software. The cut-off points of each indicator were considered according to the definition of low, medium, and high risks [16]. The definitions for each indicator as well as their cut-off points are explained in the following:

Current tobacco use

- Percentage of students who are currently smoking (smoked cigarettes on at least 1 day during the 30 days before the survey)
- Percentage of students who are currently using any tobacco products (used any tobacco products on at least 1 day during the 30 days before the survey)
- Risk Scoring: < 7 (Low); 7–15 (Medium); ≥ 16(High)

Current alcohol use

- Percentage of students who are currently abusing alcohol (at least one drink of alcohol for at least one day during the 30 days before the survey)
- Risk Scoring: < 20 (Low); 20–39 (Medium); ≥ 40 (High)

Overweight or obesity

- Percentage of students who were overweight (> + 1SD from median for BMI by age and sex)
- Percentage of students who were obese (> + 2SD from median for BMI by age and sex)
- Risk Scoring: < 10 (Low); 10–19 (Medium); ≥ 20 (High)

Physical activity

- Percentage of students who were physically active for at least 30/60 min per day on 5/7 days during the 7 days before the survey

Physical inactivity

- Percentage of not engaging students who were physically active for at least 30/60 min per day on 5/7 days during the 7 days before the survey
- Risk Scoring: < 50 (Low); 50–69 (Medium); ≥ 70 (High)

## Results

### Phase 1- comparison of the surveys

All EMR countries, except Somalia, Iran, and Saudi-Arabia implemented at least one round of GSHS. In this region, out of 22 (86%) countries, 19 implemented GSHS once following the international protocol during 2001–2018. The questionnaires used were those suggested by the international guidelines. The surveys implemented in Iran and Saudi-Arabia, which were a part of the national health surveillance system, were modified versions of the GSHS. In Iran, this modified survey, called CASPIAN study, was implemented in five rounds from 2004 to 2016 [17–25]. In Saudi-Arabia, one round of Jeeluna Study was conducted in 2011–2012 [26]. The age of the target group of these two studies differed from those of GSHSs implemented in other EMR countries (Saudi-Arabia: between 10 and 19 years and Iran: between 6 and18 years). No results were found for the implementation of any international or national study in Somalia. Table 1 shows the survey modules examined in EMR countries. Additional file 1: Appendix 1 represents the list of available publications on GSHS for each EMR country.

**Table 1:** Surveys’ modules examined in EMR countries Table

No	Module
36	Dietary behaviors and overweight
27	Alcohol and other drug use
37	Hygiene
32	Mental health
35	Physical activity
37	Protective factors
31	Tobacco use
35	Violence and unintentional injury
1	Sexual behaviours that contribute to HIV infections
1	Other Sexually transmitted infections (STIs)
1	Unintended pregnancies

Table 2 shows the included surveys’ characteristics, including year(s) of implementation, sample size, and response rate percentage. The maximum sample sizes were those of Iran (21,111 students in 2004) and the United Arab Emirates (15,790 students in 2005). In addition, the minimum sample size belonged to Yemen in 2008 (1175 students). The highest and lowest response rates were for Jordan (99.8%) in 2007 and Saudi-Arabia (62.25%) in 2012, respectively.

**Table 2:** Characteristics of the included surveys

Country		Survey’s Name	Implementation Year	Sample Size (Students)	Reponses Rate (%)
Jordan		GSHS	2004	2457	95.0
			2007	2197	99.8
Afghanistan		GSHS	2014	2579	79.0
Iran		CASPIAN	2004	21,111	–
			2007	9171	–
			2010	5570	–
			2013	13,486	90.6
			2016	14,274	99.0
United Arab Emirates		GSHS	2005	15,790	89.0
			2010	2581	91.0
			2016	5849	80.0
Bahrain		GSHS	2016	7141	89.0
Pakistan		GSHS	2009	5192	76.0
Tunisia		GSHS	2008	2870	83.0
Djibouti		GSHS	2007	1777	83.0
Sudan		GSHS	2012	2211	77.0
Syrian Arab Republic		GSHS	2010	3102	97.0
Iraq		GSHS	2012	2038	88.0
Saudi Arabia		Jeeluna	2012	12,121	62.2
Oman		GSHS	2005	2979	97.0
			2010	1606	89.0
			2015	3468	92.0
Qatar		GSHS	2011	2021	87.0
Kuwait		GSHS	2011	2672	85.0
			2015	3637	78.0
Palestine	Gaza	GSHS	2010	2677	95.0
	UNWRA^a^ Gaza			2122	95.0
	UNWRA Lebanon			2187	96.0
	UNWRA Syrian			2120	94.0
	UNWRA West Bank			2015	90.0
	West Bank			1908	94.0
Lebanon		GSHS	2005	5115	88.0
			2011	2286	87.0
			2017	5708	82.0
Libya		GSHS	2007	2242	98.0
Morocco		GSHS	2006	2670	84.0
			2010	2924	92.0
			2016	6745	91.0
Egypt		GSHS	2006	5249	87.0
			2011	2568	85.0
Yemen		GSHS	2008	1175	82.0
			2014	2655	75.0

^a^United Nations Relief and Works Agency for Palestine Refugees (UNRWA)

### Phase 2-comparison of the surveys’ results

Table 3 presents the risk levels of the non-communicable diseases’ risk factors.

**Table 3:**
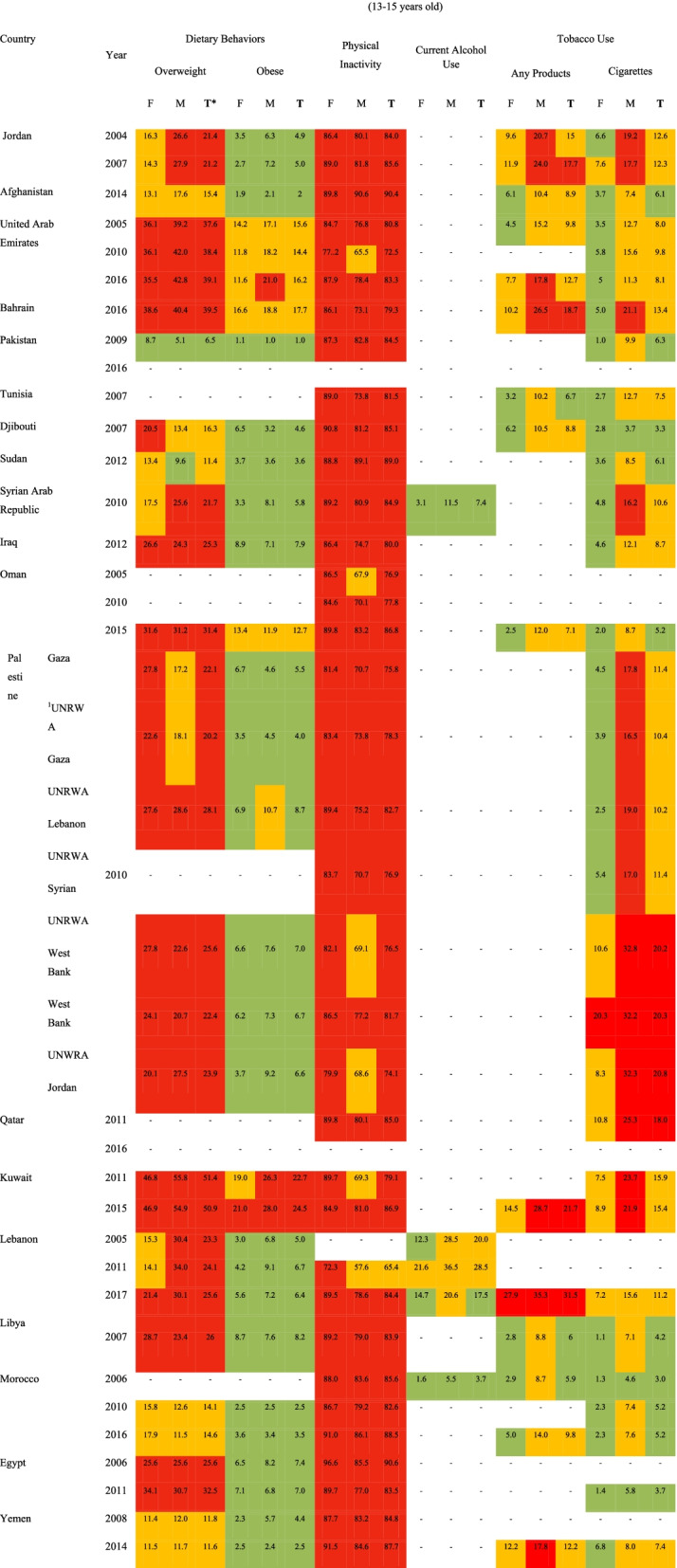
Risk levels of the non-communicable diseases’ risk factors among students aged 13–15 years in EMR counties (percent)

^1^United Nations Relief and Works Agency for Palestine Refugees (UNRWA)

*T Total, *M* Male, *F* Female

### Tobacco use

Risk levels for tobacco usage were define as follows:

**Table:** 

16% or above	7–15%	Below 7%

As shown in Table 3, male adolescents aged between 13- and 15-years old smoke cigarettes or any type of tobacco products at higher rates compared to the female ones at the same age. The highest prevalence rate of cigarette smoking in this age group among males was 25% in Qatar (2011) followed by Palestine (24% in 2010). The lowest prevalence rate of smoking cigarettes at this age group was reported as approximately 1% in females in Libya (2007), Morocco (2006), and Egypt (2011). The highest prevalence rates of using any type of tobacco products in this age group were seen in male adolescents living in Lebanon (2017) and Kuwait (2015), as 35 and 29%, respectively. As well, the lowest prevalence rate was 3% among females in Tunisia (2007), Libya. (2007), and Morocco (2006).

The prevalence rate of total tobacco usage (cigarettes and any type of tobacco products) among students aged between 13 and 15 years old ranged was 18% in Qatar (2011) and 3% in Morocco (2003). The highest prevalence rate for smoking any type of tobacco products in this age group was 31.5% in Lebanon (2017) and the lowest was 6% in Libya (2007) and Morocco (2006).

### Physical activity

Risk levels for physical inactivity were defined as follows:

**Table:** 

70% or above	50–69%	Below 50%

The results shown in Table 3 indicated that the prevalence of physical activity is relatively low among adolescents in the EMR region.

### Inappropriate dietary behaviors (obesity and overweight)

Risk levels for both obesity and overweight were defined as follows:

**Table:** 

20% or above	10–19%	Below 10%

As shown in Table 3, the prevalence of overweight is relatively high in both female and male adolescents. The highest prevalence rates of overweight and obesity were reported in males in Kuwait (56 and 28%, respectively) and the lowest levels were reported in Pakistan (5 and 1%, respectively). The overall prevalence of overweight, obesity, and physical inactivity were observed as follows: Kuwait has the highest and Pakistan has the lowest prevalence rates of overweight. The prevalence rate of obesity ranged from 1% in Pakistan to 23.6% in Kuwait. The highest prevalence of physical inactivity was reported in Egypt (90.6%) and Afghanistan (90.4%) respectively. Of note, the lowest prevalence rate was observed in Lebanon (65.4%).

### Alcohol consumption

Risk levels for alcohol consumption were defined as follows:

**Table:** 

40% or above	20–39%	Below 20%

The prevalence of alcohol consumption was reported only for three countries, including Lebanon, Morocco, and Syria. In all these three countries, alcohol consumption was higher among men.

As Table 4 shows, among the students aged between 13 and 17 years old, the highest and lowest overweight prevalence rates were reported in Kuwait (52.9%) and Iran (4.3%). In addition, the highest and lowest obesity prevalence rates were reported in Kuwait (28.2%) and Yemen (1.7%), among male adolescents, respectively. The prevalence of physical activity was relatively low among adolescents aged 13–17 years in the region. The highest and lowest physical inactivity prevalence rates in this age group were in Afghanistan (90.7%) and Bahrain (80.5%), respectively. The highest prevalence of cigarettes smokers and any type of tobacco products in this age group was in Kuwait (28.2%) and Lebanon (40.9%), among male adolescents, respectively.

**Table 4:**
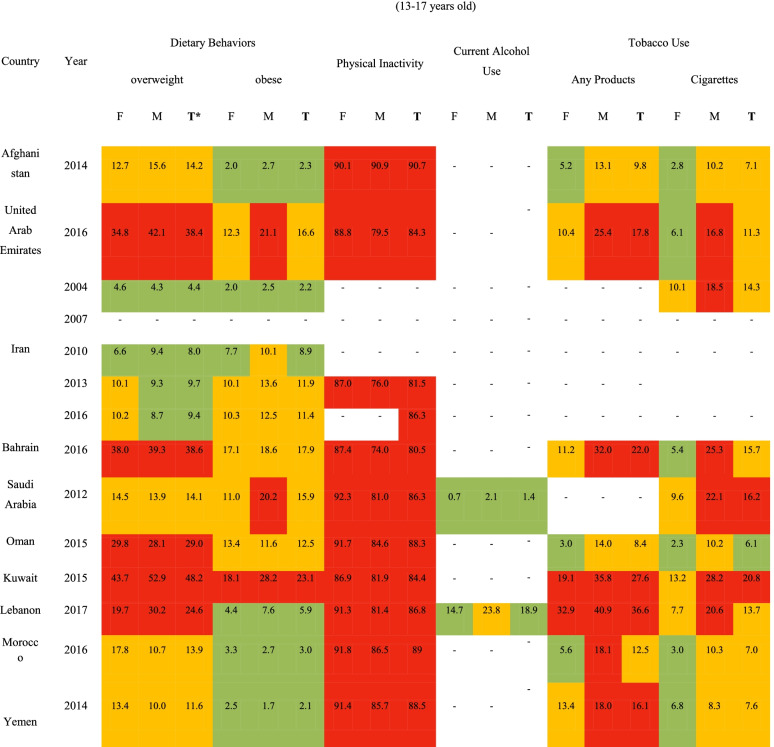
Risk levels of the non-communicable diseases’ risk factors among students aged 13–17 years in EMR counties (percent)

*TTotal, *M* Male, *F* Female

In this study, 37 reports and 10 articles were selected and included to obtain an overall estimation for each indicator by the meta-analysis. The total number of the study participants was 201,795. The heterogeneity statistics for all the indicators was obtained as approximately I^2^ > 98 by combining the results and calculating the overall estimation using a random effect model. Accordingly, this indicated that the results of the studies were extremely heterogeneous. Table 5 shows the total prevalence of overweight, obesity, physical inactivity, and tobacco use (any products and cigarettes) by the two age groups in the EMR. Sex subgroup level analysis was also performed to reduce the existing heterogeneity in results and the heterogeneous results were reported to be high yet (nearly I^2^ > 99).

**Table 5:** Result of meta-analysis and heterogeneity on prevalence of overweight, obesity, physical inactivity, tobacco use (any products & cigarettes) by age group (13–15 & 13–17 years old)

Overall (all EMR countries)						
Age group	Result	Dietary Behaviors		Physical Inactivity	Tobacco Use	
		overweight	obese		Any Products	Cigarettes
**13–15**	pooled estimate prevalence (95% CI)	24.5 (20.6–28.8)	7.3 (5.4–9.5)	82.4 (80.7–84.1)	14.3 (10.53–18.67)	9.6 (8.1–11.3)
	I^2^	99.6	99.4	98.3	99.5	98.7
	*P*value^*^	*P* < 0.001				
**13–17**	pooled estimate prevalence (95% CI)	19.8 (13.2–27.3)	9.7 (6.2–14.0)	86.1 (84.1–87.9)	17.8 (11.8–24.7)	11.5 (9.41–13.89
	I^2^	99.8	99.7	98.3	99.6	99.0
	P-value^*^	*P* < 0.001				

*p-value for Chi2 statistic for heterogeneity

## Discussion

This study aimed at examining the implementation status of GSHS in the EMR, comparing prevalence rates of the NCD risk factors, including, alcohol and tobacco use, obesity and overweight (inappropriate dietary behaviors), and physical activity among EMR countries as well as estimating an overall value for each indicator among adolescents aged between 13 and 15 and between 13 and 17 years old.

GSHS has been performed widely across EMR. However, the implemented surveys considerably differed in terms of sample sizes. The CASPIAN study conducted in Iran reported the highest sample size among the other implemented surveys. This review showed that EMR countries have not implemented GSHS in a harmonized manner, which can make it difficult to compare the relative indicators among member states over time [5]. Of note, most of the internationally recommended modules were considered by implemented surveys. However, some subjects such as high-risk sexual behaviors, alcohol and drug use, and reproductive health were not considered in the included studies, mainly due to the cultural factors. Therefore, their prevalence rates were not reported in this study.

The results for the two age groups of between 13 and 15 and between 13 and 17 years old are separately presented in the present study. The prevalence rate of physical inactivity in both age groups was estimated to be more than 80% (*P* < 0.001). In addition, the risk level was high in most years. The lowest prevalence rate was reported among adolescents aged between 13- and 17-years old living in Afghanistan in 2014. In all the countries, except Sudan and Afghanistan, the prevalence of physical inactivity was higher in male adolescents in comparison to that of female ones. The prevalence rate of overweight in the region in both age groups was high as well (24.5 and 19.8%, respectively). The results indicate that countries in where students are more physically inactive are more likely to have higher prevalence of overweight and obesity. Nowadays, more than 80% of the world’s adolescent population is insufficiently physically active [27]. Policies to reduce insufficient physical activity are being implemented in only 56% of WHO Member States in 2013 [28, 29]. Moreover, similar to other countries in the world, the EMR countries had no considerable progress. In this regard, a reduction of at least 10% in physical inactivity is recommended up to 2025 [28, 30]. Additionally, because of its high prevalence, it should be regarded as a high priority public health problem in EMR countries. Some factors that may contribute to the high prevalence of this factor in the EMR are the followings: lack of access to recreational facilities, lack of outdoor spaces (like parks), inappropriate public transportation, and social and cultural norms in the region. Females in some countries of the region mostly face some personal and social barriers to be engaged in physical activities. Therefore, it is highly recommended to consider country-specific sociocultural and environmental factors affecting physical activity in the design of appropriate interventions. Our results indicate that the higher prevalence of overweight among females is most likely due to their lower physical activity compared to males. The highest and lowest prevalence rates of overweight and obesity were reported in Kuwait and Pakistan, respectively. In another systematic review, the physical inactivity status among Arabic-speaking countries was examined in 2018, its prevalence was reported to be very high, which is similar to our results [31]. It was shown that school-based interventions can have important potentials for both obesity prevention and physical activity promotion among school-aged children and adolescents. In this regard, strategies proposed for promoting behavioral change need to take the strong role of the family supports into the account.

In the EMR, the prevalence of tobacco use was higher in male adolescents than female. The highest and lowest prevalence rates were reported in Lebanon (31.5%) and Morocco (3%) respectively. Furthermore, the overall prevalence of tobacco usage in the region was reported as 14.3% among 13–15 years old age group and as 17.8% among 13–17 years old age group. Similar to the other parts of the world, rates of tobacco usage among adolescents and young people are increasing in the EMR region as well [32]. Resolution EM / RC64 / 5, was adopted by the 61st session of the Regional Committee for the Eastern Mediterranean in 2017, which urged member states to implement the effective interventions on the prevention and control of the non-communicable diseases [33].

It may be difficult to achieve all SDG 3 targets in the EMR up to 2030. The high prevalence of the risk factors of NCDs among adolescents in this region demonstrated that interventions aiming at reducing risk factors associated with NCDs, have not achieved the expected results so far. It is highly recommended to include culturally relevant, evidence-based trainings in the school curricula. Correspondingly, this could be considered as one of the best opportunities to correct the lifestyle of this age group. As well, it is necessary to provide sufficient facilities for the purpose of improving the physical activity level of young people as well as promoting healthy eating behaviors in schools. This issue should be considered when the allocation of the educational budgets in this region.

To prioritize the interventions needed to tackle the NCDs risk factors based on our results, physical inactivity was found as the most important modifiable risk factor that should be addressed by EMR countries in the near future. Considering the high prevalence of youth smoking in the EMR, it is suggested that tobacco usage in adolescents and its contributing factors be explored further in the region. A coalition of youth advocates for health in the EMR was launched in 2017 and the issue needs to be emphasized in future meetings of the coalition.

Our analysis has some important limitations. First, the quality of secondary data from surveys can affect the final results. Second, the age ranges of the countries surveys vary that should be taken into account while interpreting the results.

## Conclusion

Since implementation of GSHS survey received considerable attentions of EMR member states, it can be concluded that the risk factors of NCDs in the adolescents are reasonably monitored in the region. As a further step, a harmonized survey could be designed in the region, in order to facilitate the comparison of the results across the countries. Although the NCD risk factors are being monitored through GSHS surveys, many targets related to the risk factors have not been achieved in the region. It is critical that comprehensive policies be further designed to address adolescent health issues and to measure the impact of any intervention. Furthermore, an appropriate surveillance system should be designed to monitor the current situation of the NCD risk factors among young people. However, survey’s implementation per se cannot guarantee the proper monitoring of the NCD risk factors. Further the capacity of EMR countries for surveillance of NCDs, their risk factors and determinants, as well as utilizing the obtained results in NCD programs’ monitoring and evaluation should be strengthened. Among the examined risk factors in this study, insufficient physical activity in adolescents, which needs an instant action to prevent and control, should be promptly considered as a major public health problem by all countries.

## Supplementary Information

**Additional file 1:** **Appendix 1.**

## Data Availability

The datasets generated and/or analyzed during the current study are not publicly available due to they don’t not provide more necessary information, but are available from the corresponding author on reasonable request.

## Abbreviations

NCDs: Non-communicable diseases
GSHS: Global School-based Student Health Survey
EMR: Eastern Mediterranean Region
WHO: The World Health Organization
CDC: Centers for Disease Control and Prevention
