# Supplementary material for: Global school-based student health survey: country profiles and survey results in the eastern Mediterranean region countries
Source: BMC Public Health. 2022 Jan 19;22:130. doi: 10.1186/s12889-022-12502-8 (PMC8767753; doi:10.1186/s12889-022-12502-8)
Supplement: Supplementary file 1 — Additional file 1: Appendix 1. [file 12889_2022_12502_MOESM1_ESM.docx]

**Appendix**

**[Appendix](https://dictionary.abadis.ir/entofa/a/appendix/) 1. List of GSHS-related publications in the countries of the Eastern Mediterranean region**

| **Country** | | **Year** | **Questionnaire** | **Factsheet** | **Data & Documentation** | **Report** |
| --- | --- | --- | --- | --- | --- | --- |
| Jordan | | 2004 | * | * | * | * |
|  |  | 2007 | * | * | * | * |
| Afghanistan | | 2014 | * | * | * | - |
| United Arab Emirates | | 2005 | * | * | * | * |
|  |  | 2010 | * | * | * | - |
|  |  | 2016 | * | * | - | - |
| Iran | | 2004 | - | - | - | - |
|  |  | 2007 | - | - | - | * |
|  |  | 2010 | * | - | - | * |
|  |  | 2013 | * | - | * | * |
|  |  | 2016 | * | - | * | * |
| Bahrain | | 2016 | * | * | * | - |
| Pakistan | | 2009 | * | * | * | - |
|  |  | 2016 | * | - | - | - |
| Tunisia | | 2007 | * | * | * | - |
| Djibouti | | 2007 | * | * | * | * |
| Sudan | | 2010 | * | * | * | - |
| Syrian Arab Republic | | 2010 | * | * | * | - |
| Iraq | | 2012 | * | * | * | - |
| Saudi Arabia | | 2012 | - | - | - | - |
| [Oman](https://www.who.int/ncds/surveillance/gshs/oman/en/) | | 2005 | * | * | * | * |
|  |  | 2010 | * | * | * | * |
|  |  | 2015 | * | * | * | - |
| Palestine | Gaza | 2010 | * | * | * | - |
|  | ^^[[1]](#footnote-1)^^UNWRA  Gaza |  |  | * | * |  |
|  | UNWRA  Lebanon |  |  | * | * |  |
|  | UNWRA  Syrian |  |  | * | * |  |
|  | UNWRA  West Bank |  |  | * | * |  |
|  | West Bank |  |  | * | * |  |
|  | UNWRA  Jordan |  |  | * | * |  |
| Qatar | | 2011 | * | * | * | - |
|  |  | 2016 | * | - | - | - |
| Kuwait | | 2011 | * | ^*^ | * | * |
|  |  | 2015 | * | * | * | - |
| Lebanon | | 2005 | * | * | * | * |
|  |  | 2011 | * | * | * | - |
|  |  | 2017 | * | * | - | - |
| Libya | | 2007 | * | * | * | * |
| Morocco | | 2006 | * | * | * | - |
|  |  | 2010 | * | * | * | - |
|  |  | 2016 | * | * | - | - |
| Egypt | | 2006 | * | * | * | - |
|  |  | 2011 | * | * | * | - |
| Yemen | | 2007 | * | * | * | - |
|  |  | 2014 | * | * | * | - |

1. United Nations Relief and Works Agency for Palestine Refugees [↑](#footnote-ref-1)
